# Newborn Rabbit Perception of 6-Odorant Mixtures Depends on Configural Processing and Number of Familiar Elements

**DOI:** 10.1371/journal.pone.0107560

**Published:** 2014-09-23

**Authors:** Sébastien Romagny, Thierry Thomas-Danguin, Gérard Coureaud

**Affiliations:** Centre des Sciences du Goût et de l'Alimentation (Research Center for Taste and Feeding Behaviour), Dijon, France; Université Lyon, France

## Abstract

Perception of odors, i.e. usually of mixtures of odorants, is elemental (the odorants' odor qualities are perceived in the mixture) or configural (the odor quality of the mixture differs from the one of each odorant). In human adults, the Red Cordial (RC) mixture is a configurally-processed, 6-odorant mixture. It evokes a red cordial odor quality while none of the elements carries that odor. Interestingly, in newborn rabbits, the same RC mixture is weak configurally perceived: the newborns behaviorally respond to all the elements after conditioning to the whole mixture, but not to the mixture after conditioning to a single element. Thus, they perceive in the RC mixture both the odor quality of the RC configuration and the quality of each element. Here, we aimed to determine whether this perception is modulated by quantitative (number of elements) and/or qualitative bits of information (nature of elements) previously learned by the animals. Newborns were conditioned to RC sub-mixtures of different complexity and composition before behavioral testing to RC. Pups generalized their sucking-related response to RC after learning at least 4 odorants. In contrast, after conditioning to sub-mixtures of another 6-odorant mixture, the elementally perceived M^V^ mixture, pups responded to M^V^ after learning one or two odorants. The different generalization to RC and M^V^ mixtures after learning some of their elements is discussed according to three hypotheses: i) the configural perception of RC sub-mixtures, ii) the ratio of familiar/unfamiliar individual information elementally and configurally perceived, iii) the perception of RC becoming purely elemental. The results allow the first hypothesis to be dismissed, while further experiments are required to distinguish between the remaining two.

## Introduction

Natural odors contain several odorants that are thus experienced in mixtures [1]–[3]. Dealing with mixtures, the olfactory system functions in two alternative, but not systematically exclusive, ways. When an organism detects and responds to the odorants included in a mixture, i.e. when the mixture smells like the elements, its perception is elemental. Conversely, when the odor quality of the mixture differs from that of the odorants and gives rise to a novel perceptual odor quality, the perception is configural [4]. The configural perception is “robust” when the organism is unable to distinguish any element in the mixture, which is the case in the perception of coffee or chocolate odor qualities in humans [5]. The configural perception is “weak” when the organism perceives the new percept in addition to the odors of the elements [4].

Human adults perceive the binary AB mixture of ethyl isobutyrate (odorant A, strawberry odor) and ethyl maltol (odorant B, caramel odor) as more pineapple-like compared to the single odorants, a result supporting the configural perception of this mixture [6], [7]. Strikingly, when newborn rabbits are conditioned once to the odorant A, they do not later generalize their behavioral (sucking-related) response to the AB mixture, meaning that the odor qualities of A and AB are different [8]. This absence of responsiveness is not due to overshadowing (same results are obtained after conditioning to odorant B) nor to the perception of an unfamiliar odorant in addition to A, since the pups are able to generalize from A to an AC mixture [9]. When conditioned to the AB (or AC) mixture, newborn rabbits later respond both to odorants A and B (respectively A and C), which suggests that they perceive the AB mixture in a weak configural way and the AC mixture in a pure elemental way. Thus, in the AB mixture, newborns seem to perceive the odor of A, the odor of B, and a particular odor for the AB configuration [8], [9]. This weak configural perception of AB has been recently confirmed through a pharmacological approach showing that the neonatal memory of the AB configuration differs from the memory of the A and B elements [10], [11].

The particular perception of the AB mixture by rabbit neonates depends on the A and B chemicals, their ratio in the mixture [12] and two other factors. First, the number of learned elements: after successive conditioning to A then to B, pups generalize their response to AB [8]. Thus, the perception of only one familiar bit of information (e.g., odor A after conditioning to A) among the three contained in the AB mixture (A, B and the AB configuration) is not sufficient to trigger the response to the whole mixture, whereas perceiving two familiar bits of information (odors A and B) among the three promotes the response. Second, the pups' experience of a single element modulates the perception of the AB mixture: after repeated conditioning to A (or B), the pups respond to AB, suggesting that the mixture becomes more elementally perceived [13].

The elemental vs configural perception of binary odor mixtures and its evolution according to experience raise the question of what happens with mixtures of higher complexity. Several results highlighted that the more complex a mixture is, the less elemental is the perception [14]. For instance, squirrel monkeys better discriminate sub-mixtures of 3 or 6 than 9 or 11 components from a 12-component mixture [15]. Similarly, human adults are not able to recognize the different odorants in a mixture as soon as it includes more than 4 components [16]. They cannot even recognize any odorant in a mixture of 16 components [17] and perceive a “white” odor in mixtures of 30 iso-intense odorants which, as reported by the authors, span the odorants' chemical space [18]. Despite its ecological relevance, few studies have focused on the role of experience on the perception of complex mixtures. In human adults, even after training, the limit of perceptual analysis seems to remain at 4 elements [19] suggesting that when the mixture's complexity increases, perceptual plasticity is less open to the influence of experience. Here, we investigated this point in rabbit pups, and determined how learning of odorants in sub-mixtures of various complexity influences the perception of the sub-mixtures but also, and more importantly, influences the perception of two senary mixtures containing the learned odorants among others.

To that goal, we used the 6-component RC mixture previously identified as configurally perceived both in newborn rabbits and human adults. Humans perceive the odor of red cordial in that mixture whereas none of its elements carries that odor quality [6], [20]. In the rabbit, after conditioning to RC, the pups respond to each of its odorants; however, after conditioning to one odorant, they do not respond to RC. Therefore, they are likely to perceive weak configurally the RC mixture, i.e. to perceive a particular odor quality (configuration) in addition to the specific quality of each component. Such perception does not occur for any senary mixture: after conditioning to vanillin (one of the RC components), pups respond to a M^V^ mixture including vanillin and five other components absent from RC [20]. Thus, pups can process mixtures of 6 components either configurally (RC) or purely elementally (M^V^) depending on the mixed odorants. Here, we evaluated whether the learning of more than one element contained in these two mixtures, and/or the nature (odor quality) of the learned elements, influence their perception. Newborns were conditioned to sub-mixtures of 2 to 5 components before behavioral testing both to the sub-mixtures, their odorants and the RC mixture. Based on results previously obtained with the AB and AC binary mixtures [8], [9], we hypothesized that pups would generalize their response to the RC mixture when they have learned more than 50% of its odorants (Experiment 1), but that the number of learned odorants would not influence the responsiveness to the elementally perceived M^V^ mixture (Experiment 2). Additionally, we determined whether the RC configural mixture is a combination of sub-mixtures by themselves configurally processed, i.e. of sub-configurations (Experiment 3).

## Materials and Methods

### Animals and housing conditions

Males and females New Zealand rabbits, *Oryctolagus cuniculus* (*Linnaeus*) (Charles River; L'Arbresle, France), from the Centre de Zootechnie de l’Université de Bourgogne (Dijon) were kept in individual cages, and a nest box (0.39 m×0.25 m×0.32 m) was added on the outside of the pregnant females' cages 2 days before delivery (the day of delivery was considered as postnatal day 0). To equalize the nursing experience of the pups, all the females had access to their nest between 11:30 and 11:45 a.m. This procedure made it possible to follow the brief (3–4 min), usually daily nursing of the pups [21]. The animals were kept under a constant 12h:12h light:dark cycle (light on at 07:00 a.m.) with ambient air temperature maintained at 21–22°C. Water and pelleted food (Lapin Elevage 110, Safe, France) were provided *ad libitum*. A total of 375 newborns from 82 females were used in the study.

We strictly followed the local, institutional and national rules (French Ministries of Agriculture, and of Research and Technology) regarding the care and experimental use of the animals. Thus, all experiments were carried out in accordance with ethical rules enforced by French law, and were approved by the Ethical Committee of the University of Burgundy (Dijon, France; no. 5305).

### Odorants and mixtures

The odorants were all purchased from Sigma-Aldrich (Saint-Quentin Fallavier, France). Stock solutions of the odorants were prepared in ethanol (anhydrous, 99.9% Carlo Erba, France), then diluted in purified water (MilliQ System, Millipore, Molsheim, France) and mixed to reach the target concentration and ratio of odorants in mixtures. The mixtures, sub-mixtures and single odorants were finally diluted in a solvent made of purified water and a maximum of 0.1% of absolute ethanol. At this concentration level, ethanol does not carry significant odor for rabbit pups: it is certainly not detectable thus not active at all on their behavior [8].

A first mixture, the RC mixture, was composed of 6 components: vanillin (odorant V; CAS # 8014-42-4), frambinone (F; CAS # 5471-51-2), isoamyl acetate (IA; CAS # 123-92-2), β-ionone (B; CAS # 79-77-6), ethyl acetate (EA; CAS # 141-78-6) and damascenone (D, CAS # 23696-85-7). Stock solutions of the odorants were prepared at 1% (w/w) in ethanol. Sinding et al. [20] showed that these single odorants did not spontaneously trigger sucking-related response in rabbit pups, and can be therefore considered as behaviorally neutral stimuli before conditioning. The odorant proportions in the RC mixture were respectively 41.8/41.8/5.0/4.3/4.3/2.8% (final concentrations 3.3/3.3/0.39/0.34/0.34/0.22×10^−6^ g/ml) for V/F/IA/B/EA/D; at these proportions, the mixture elicits the configural perception of a red cordial odor in human adults, and a weak configural perception in rabbit neonates [20].

All the components of the RC mixture were also used as single stimuli or paired with the unconditioned stimulus (the mammary pheromone; MP) described below. In these cases, the single odorant concentrations were the same as the concentrations of each odorant in the mixture.

Among the 56 possible sub-mixtures of RC including 2 to 5 components, we have chosen 12 sub-mixtures based on the previous results of a free sorting task carried out in human adults [20]. According to this study, the odor of the mixture was different from the odors of the components, but some of them were perceptually closer in terms of odor quality. Thus, we chose for the present study 4 sub-mixtures composed with the components which were perceived by humans as being the more similar to the RC mixture: V-IA, V-IA-F, V-IA-F-D, V-IA-F-D-B, and 4 mixtures formed with the components which were perceived by humans as being the less similar to the RC mixture: EA-B, EA-B-D, EA-B-D-F, EA-B-D-F-IA. Moreover, by choosing two supplementary sub-mixtures of 3 components, V-IA-D and EA-B-F, and two other mixtures of 4 components, V-IA-D-B and EA-B-F-IA, we ensured that pups were exposed to all the RC odorants and to sub-mixtures which did not share the same arrangement of components. Concentrations of components in the sub-mixtures were the same as for single components. As a control, we checked for the iso-intensity of all the stimuli with the help of a human panel (n = 18). Moreover, the overall volatile organic compounds concentration was measured for all samples using a photoionization detector (static headspace at 23°C). We thus ensured that odorant concentration in the headspace was not the driving factor of pups' responsiveness (a Pearson's correlation between pups' responsiveness and volatile organic compounds concentration was not significant: r = 0.35, p = 0.26).

The M^V^ mixture was another mixture of 6 odorants. It shared one component with the RC mixture: vanillin. The other components were: n-butanol (N, CAS # 71-36-3), α-pinene (P, CAS # 80-56-8), eucalyptol (E, CAS # 470-82-6), linalool (L, CAS # 78-70-6) and cis-3-hexen-1-ol (C3H, CAS # 928-96-1). Stock solutions of the components were at 30/30/30/5/1/1% (w/w in ethanol) respectively for V/N/P/E/L/C3H. The M^V^ mixture included 16.7 µl of each stock solutions diluted in 100 ml of water to reach final concentrations of 3.9/3.9/3.9/0.66/0.13/0.13×10^−5^ g/ml respectively for V/N/P/E/L/C3H. At this ratio, the mixture is perceived in an elemental way by rabbit pups (and by human adults [20]). Four sub-mixtures of M^V^ with increasing complexity were also chosen: a sub-mixture of 2 and a sub-mixture of 3 components which did not overlap in terms of odorants (N-L and E-P-C3H) to avoid that pups respond to a particular quality of the odorants, and two sub-mixtures of 4 and 5 components including the previous sub-mixture of 3 plus other odorants of the M^V^ mixture (E-P-C3H-N, E-P-C3H-N-L). Since Sinding et al. [20] showed that pups perfectly responded to M^V^ after conditioning to any one of its components, we suspected that they would highly respond to the present M^V^ sub-mixtures. All the components of M^V^ were also used as single stimuli. In these cases, the single odorant concentrations were the same as those in the mixture. In a preliminary experiment, we showed that 2-day-old rabbits did not spontaneously respond to the M^V^ mixture nor to its components by typical orocephalic movements (responsiveness <8.3%, n = 12 and 12 pups, when respectively N, L, E and M^V^ or P, CH3, V and M^V^ were presented. The low responsiveness was equivalent to the responsiveness to the solvent; χ^2^<0.002, p>0.05).

Finally, the mammary pheromone (MP, 2-methylbut-2-enal, CAS # 1115-11-3) was used as unconditioned stimulus to induce the learning of odorants or sub-mixtures by associative conditioning. To that goal, it was used at 10^−5^ g/ml (see below and [8]–[13], [20], [22]).

### Odor conditioning

The conditioning took place in an experimental room close to the breeding room. On day 1, for each litter, a maximum of five pups were transferred from the nest to the experimental room into a box maintained at room temperature (the other pups of the litters were not used). The MP-induced conditioning was run following a procedure previously described [8]–[13], [20], [22], [23]. Immediately before the beginning of the conditioning, a cotton pad was soaked with 3 ml of the conditioned stimulus (odorant, sub-mixture or full mixture) plus the unconditioned stimulus (MP). Then, the cotton pad was held 2 cm above the pups for 5 min. Two minutes after the end of the conditioning, pups were individually marked and returned to the nest. The box containing the pups was rinsed with ethanol then filtered water (Milipore, Molsheim, France) between conditioning sessions. Conditionings occurred 1 h before the daily nursing (around 10:30 a.m.) to equalize the pups' motivational state and limit the impact of satiation on responses [24].

### Behavioral assay

The behavioral assay occurred on day 2, between 10:00 and 10:30 a.m., in the room previously used for the conditioning. It consisted in an oral activation test [8]–[13], [20], [22]. Pups were individually maintained in one gloved hand of the experimenter, allowing only head movements (gloves were not systemically changed between the testing of each pup but the putative bias induced by the contamination of the glove by the odor of a pup cannot explain the strongly contrasted responsiveness often displayed in and between the distinct experiments). The odor stimulus was presented with the other hand for 10 s on the tip of a glass rod, 0.5 cm in front of the nares. The test was positive when the stimulus elicited head-searching movements (vigorous, low amplitude horizontal and vertical scanning movements displayed after stretching towards the rod) followed usually by grasping movements (labial seizing of the tip of the glass rod). Non-responding pups displayed no response but sniffing. Each pup participated in only one experiment but was successively tested with four stimuli (five in Experiment 3), i.e. the conditioned stimulus to systematically assess the effectiveness of the conditioning and, depending on the experiments, the target mixtures, sub-mixtures, or odorants. Successive testing involved the presentation of a first stimulus to the pups from a same litter, then a second stimulus and so on, with an inter-trial interval of 120 s. The order of stimulus presentation was systematically counterbalanced from one pup to another. If a pup responded to a stimulus, its nose was softly dried before the next stimulation. The pups were immediately reintroduced to their nest after testing. To minimize litter effects, each experimental group was drawn from four or five litters, with a maximum of five pups tested per litter.

### Statistics

The pups that did not respond to any of the tested stimuli, even the conditioned one (n = 14/375), were kept in the statistical analysis to maintain individual variability in the results. The frequencies of responding pups were compared using the Cochran's Q test for paired data when same pups were tested for their response to several stimuli; if the Cochran's Q test was significant, proportions of responding pups were compared 2×2 using the McNemar's χ^2^ test. When the data were independent (different groups of pups tested to a same stimulus), the Pearson's χ^2^ test was used. Degrees of freedom are indicated when> 1. Data were considered as significantly different when the tests yielded p<0.05. The analyses were conducted using R software release 2.14.1 [25].

## Results

### Experiment 1 - Number and nature of familiar elements required to respond to the RC configural mixture

The aim of this first series of experiments was to evaluate whether rabbit pups become able to respond to the RC mixture after learning more than one component and whether the responsiveness depended on the number and/or nature of the learned components. The strategy was to condition the pups to sub-mixtures of 2, 3, 4 or 5 components of RC on day 1, and to test them, on day 2, for their responsiveness to the conditioned sub-mixture, the components of the sub-mixture and the RC mixture.

#### Experiment 1.1 - Conditioning to sub-mixtures of 2 odorants

**Figure 1:**
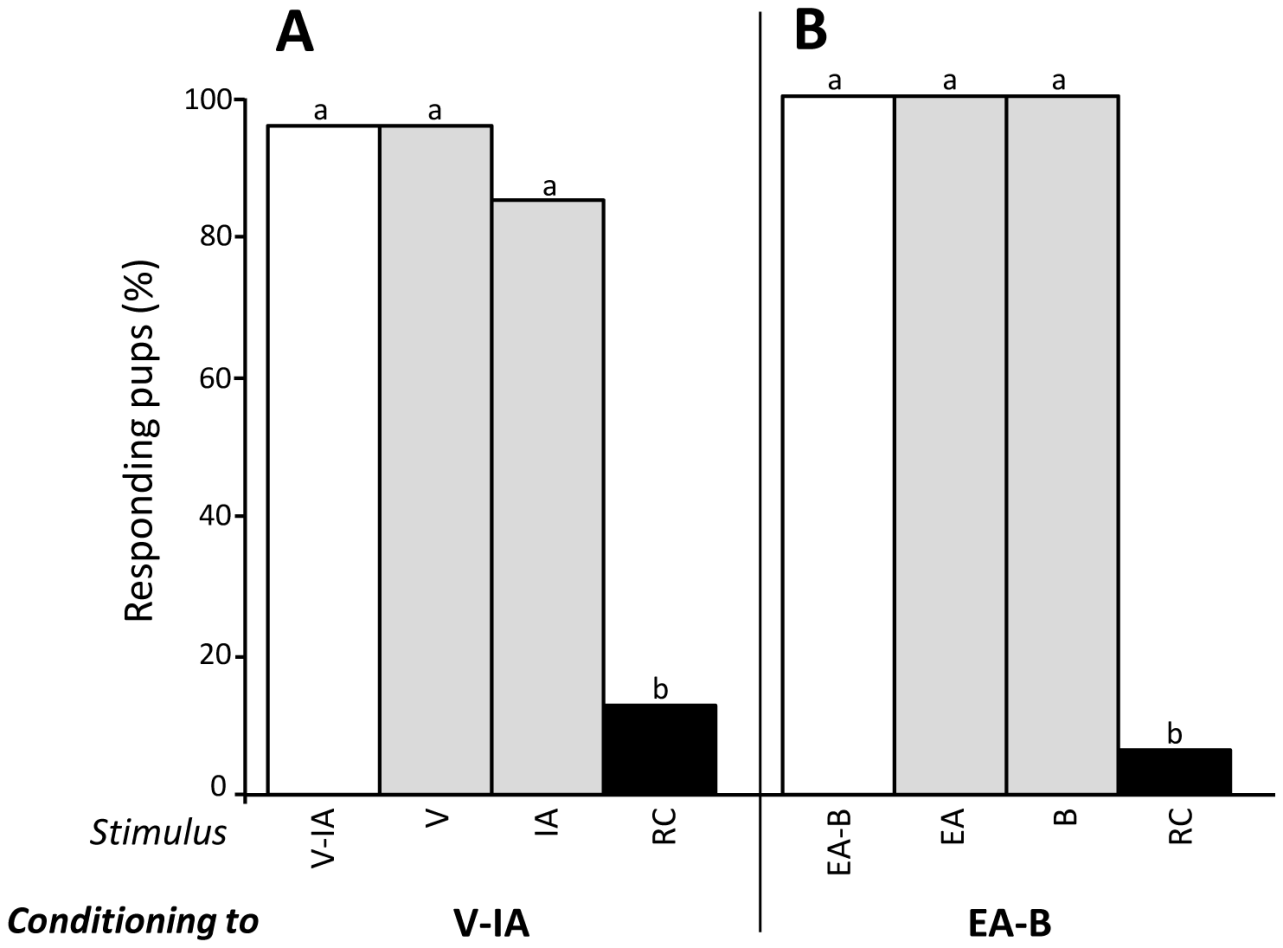
Proportions of 2-day-old rabbit pups responding in an oral activation test to (A) the V-IA sub-mixture (including the more RC-typical components for humans; white bar), the single odorants V and IA (grey bars) and the RC mixture (black bar) after conditioning to V-IA (n = 20 pups), or (B) the EA-B sub-mixture (including the less RC-typical components for humans), the single odorants EA, B and the RC mixture after conditioning to EA-B (n = 18). In each graph, statistical differences are indicated by the letters on top of the bars (p<0.05).

After conditioning to the V-IA sub-mixture (n = 20), the pups responded differently to the stimuli (Q = 43, d.f. = 3, p<0.001):>80% responded to V-IA and to each component presented separately (V or IA; χ^2^<0.5, p>0.05), while 10% only responded to the RC mixture (comparisons RC vs V-IA, V or IA: χ^2^>12, p<0.001) (Figure 1A). Thus, MP-induced conditioning promoted neonatal learning of the V-IA sub-mixture, but the acquisition of V-IA was not sufficient to induce the generalization of the response to the RC mixture. To determine whether this non-response resulted from the nature of the learned odorants, a second group of pups (n = 18) was conditioned to the sub-mixture of two other RC elements (odorants EA and B). Again, the pups' responsiveness varied significantly between the stimuli (Q = 51, d.f. = 3, p<0.001): strong (94.7%) both to the sub-mixture and each component (χ^2^<0.5, p>0.05) it was extremely weak to RC (5%; comparisons RC vs EA-B, EA or B: χ^2^ = 15.1, p<0.001) (Figure 1B).

Thus, conditioning to distinct 2-component sub-mixtures of RC was insufficient to promote the responsiveness of rabbit pups to the whole mixture.

#### Experiment 1.2 - Conditioning to sub-mixtures of 3 odorants

**Figure 2:**
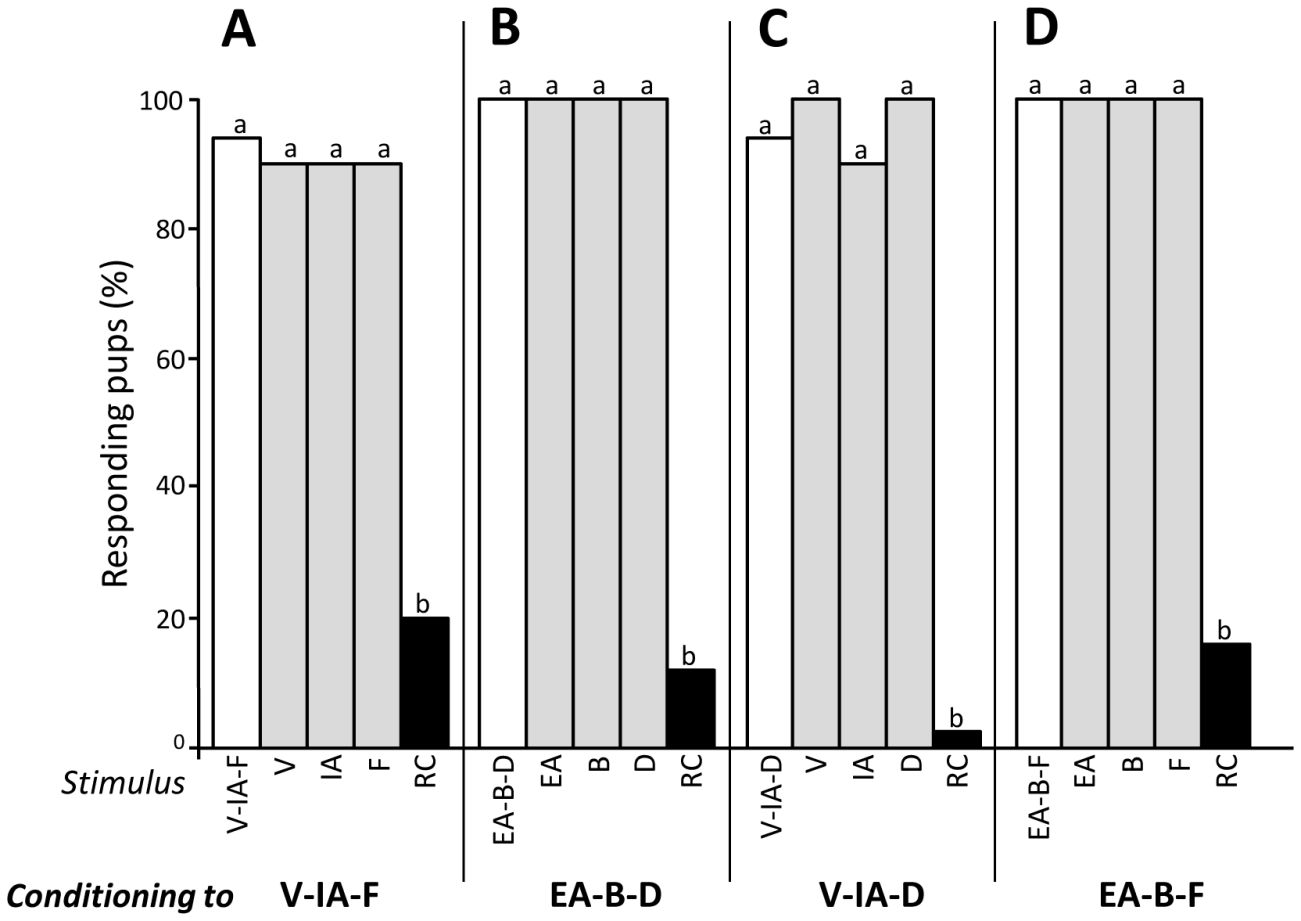
Proportions of 2-day-old rabbit pups responding in an oral activation test to (A) the V-IA-F sub-mixture (including the more RC-typical components for humans; white bar), the single odorants V, IA, F (grey bars) and the RC mixture (black bar) after conditioning to V-IA-F (n = 20 pups), (B) the EA-B-D sub-mixture (including the less RC-typical components for humans), the single odorants EA, B, D and the RC mixture after conditioning to EA-B-D (n = 17), (C) the V-IA-D sub-mixture, the single odorants V, IA, D and the RC mixture after conditioning to V-IA-D (n = 19), or (D) the EA-B-F sub-mixture, the single odorants EA, B, F and the RC mixture after conditioning to EA-B-F (n = 19). In each graph, statistical differences are indicated by the letters on top of the bars (p<0.05).

For each of the four sub-mixtures that we used, the pups' responsiveness differed among the stimuli (Q>17, d.f. = 3, p<0.001). Thus, after conditioning to V-IA-F, when tested to this sub-mixture, RC, V and IA (n = 10 pups), or to the sub-mixture, RC, IA and F (n = 10), the pups strongly (>90%) responded to the sub-mixture and its odorants, but only weakly to RC (20%; comparisons RC vs V-IA-F, V, IA or F: χ^2^>4.2, p<0.05) (Figure 2A). After conditioning to EA-B-D and testing to this sub-mixture, RC, B and respectively to EA or D (n = 8 and 9 pups), all the pups responded to EA-B-D and the single odorants, but only 10% responded to RC (RC vs EA-B-D, EA, B or D: χ^2^>4.2, p<0.05) (Figure 2B). After conditioning to V-IA-D and testing to this sub-mixture, RC, D and respectively to V or IA (n = 9 and 10 pups), more than 94% of the pups responded to V-IA-D and to its individual components, but none responded to RC (RC vs V-IA-D, V, IA or D: χ^2^>20, p<0.001) (Figure 2C). Finally, after conditioning to EA-B-F and testing to this sub-mixture, RC, F and to B or EA (n = 9 and 10 pups), all the pups responded to EA-B-F and each odorant, but only 15.7% responded to RC (RC vs EA-B-F, EA, B or F: χ^2^>14, p<0.001) (Figure 2D).

Thus, conditioning to certain sub-mixtures of three RC-elements induced the learning of the odorants and subsequent behavioral response to both the sub-mixtures and odorants, but it remained insufficient to trigger responsiveness to the RC mixture.

#### Experiment 1.3 - Conditioning to sub-mixtures of 4 odorants

**Figure 3:**
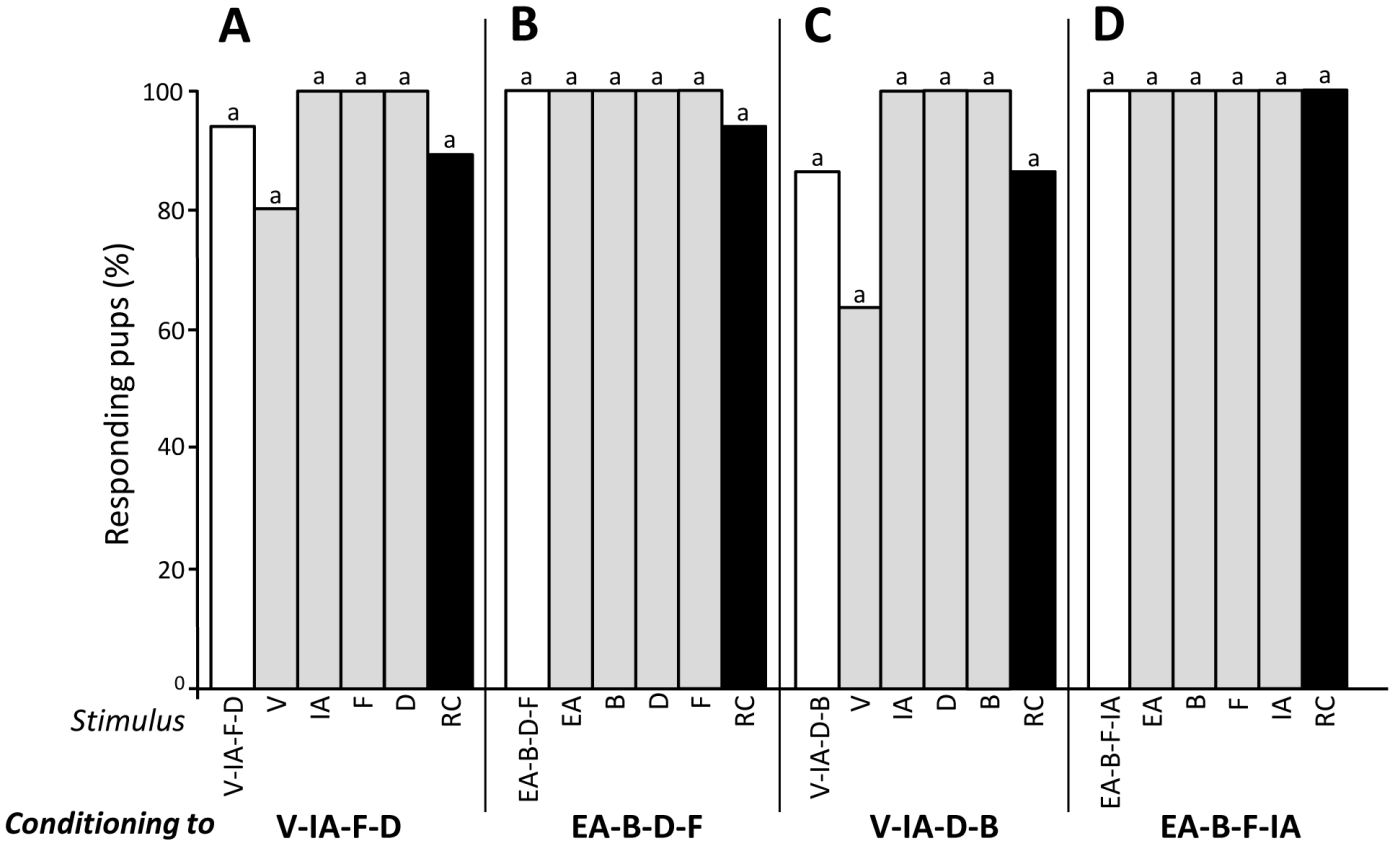
Proportions of 2-day-old rabbit pups responding in an oral activation test to (A) the V-IA-F-D sub-mixture (including the more RC-typical components for humans; white bar), the single odorants V, IA, F, D (grey bars) and the RC mixture (black bar) after conditioning to V-IA-F (n = 20 pups), (B) the EA-B-D-F sub-mixture (including the less RC-typical components for humans), the single odorants EA, B, D, F and the RC mixture after conditioning to EA-B-D-F (n = 20), (C) the V-IA-D-B sub-mixture, the single odorants V, IA, D, B and the RC mixture after conditioning to V-IA-D-B (n = 16), or (D) the EA-B-F-IA sub-mixture, the single odorants EA, B, F, IA and the RC mixture after conditioning to EA-B-F-IA (n = 20). In each group, responsiveness to the different stimuli did not differ significantly (p>0.05).

A first group of 20 newborns was conditioned to V-IA-F-D, then tested to this sub-mixture, RC, V and IA (n = 10 pups) or to the sub-mixture, RC, F and D (n = 10 pups). In each sub-group, the responsiveness was strong and similar to all the stimuli, including to RC (>80%; Q<3, d.f. = 3, p>0.05). Thus, after learning V-IA-F-D, the pups responded also to the whole RC mixture (Figure 3A). To determine the generality of this effect, 20 other pups were conditioned to another quaternary sub-mixture, EA-B-D-F, and tested to RC and the single odorants EA and B (n = 10 pups) or RC and the odorants D and F (n = 10 pups) in addition to the conditioned sub-mixture. Again, in each sub-group, pups strongly responded to all stimuli, RC included (>95%; Q<3, d.f. = 3, p>0.05) (Figure 3B).

Since the responsiveness to RC could result from the presence of the odorants F and D in the V-IA-F-D and EA-B-D-F sub-mixtures, we repeated the procedure with two other quaternary sub-mixtures devoid of the simultaneous presence of F and D. Thus, 16 and 20 newborns were conditioned either to V-IA-D-B or EA-B-F-IA, and tested either to V-IA-D-B, RC and odorants V and IA (n = 8 pups) or D and B (n = 8 pups), or to EA-B-F-IA, RC and odorants B and F (n = 10 pups) or EA and IA (n = 10 pups). Pups responded similarly to the conditioned sub-mixture, its single components, and the RC mixture (>62.5%; Q<3, d.f. = 3, p>0.05) (Figure 3C,D).

Thus, after conditioning to a quaternary sub-mixture of RC, rabbit pups were able to respond not only to the conditioned sub-mixture and its single components but also to the complete RC mixture.

#### Experiment 1.4 - Conditioning to sub-mixtures of 5 odorants

**Figure 4:**
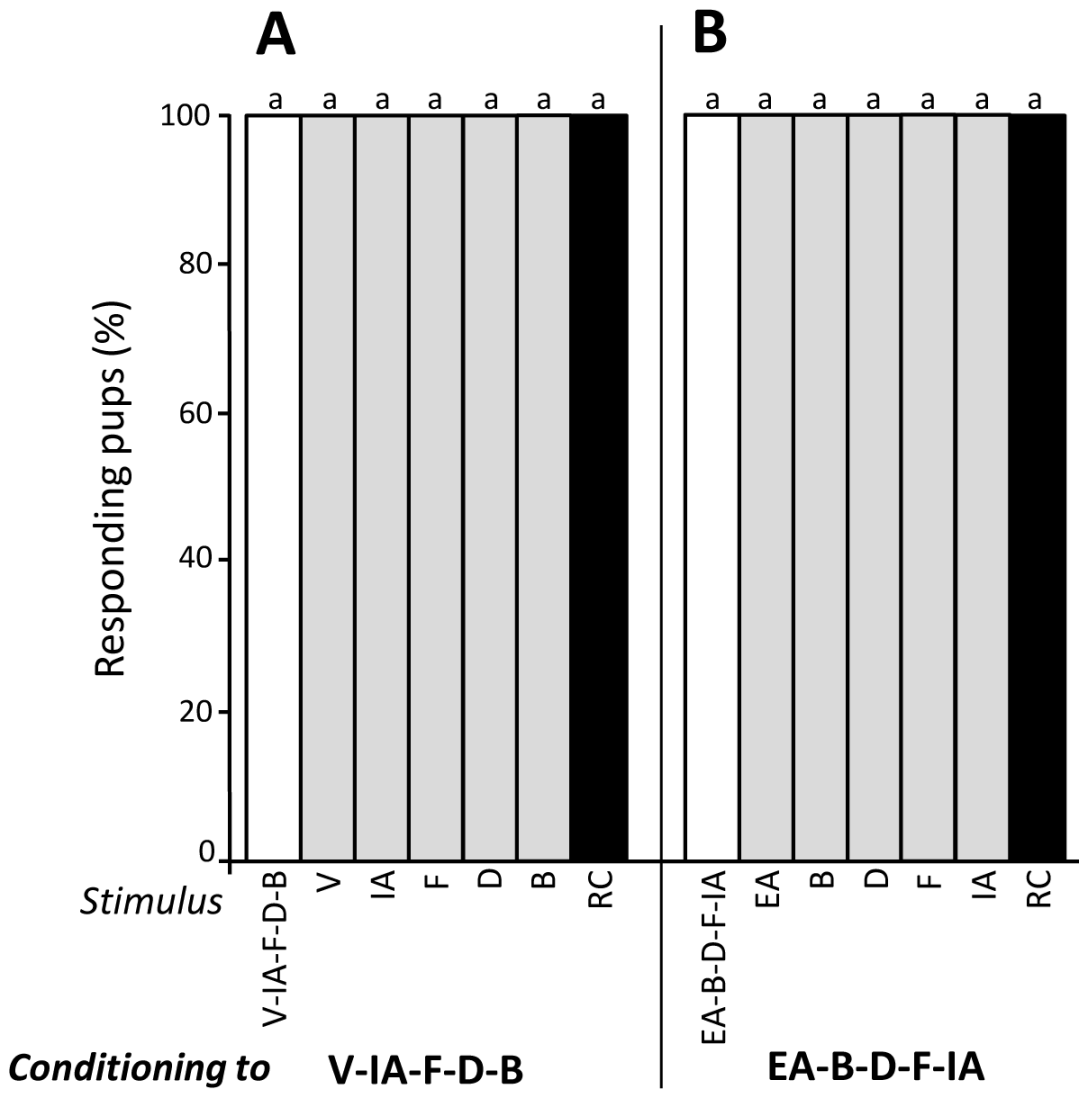
Proportions of 2-day-old rabbit pups responding in an oral activation test to (A) the V-IA-F-D-B sub-mixture (including the more RC-typical components for humans; white bar), the single odorants V, IA, F, D and B (grey bars) and the RC mixture (black bar) after conditioning to V-IA-F-D-B (n = 20 pups), or (B) the EA-B-D-F-IA sub-mixture (including the less RC-typical components for humans), the single odorants EA, B, D, F, IA and the RC mixture after conditioning to EA-B-D-F-IA (n = 20). In each group, responsiveness to the different stimuli was maximal.

In light of the results of Exp. 1.3, one might expect that the learning of 5-component sub-mixtures of RC promotes the subsequent response to the RC mixture. To assess this assumption, 2 groups of 20 newborns were conditioned either to V-IA-F-D-B or EA-B-D-F-IA, and respectively exposed, per sub-groups of 10 pups, to the first quinary sub-mixture, RC, odorants V and F or IA and B, or to the second sub-mixture, RC, odorants EA and B or D and IA. In both groups and every sub-group, the pups' responsiveness was optimal whatever the stimulus, RC included (100%, Q<0.1, d.f. = 5, p>0.05) (Figure 4A,B). The level of responsiveness to RC was then similar in pups conditioned to 4- and 5-component sub-mixtures (>84%, p>0.05 in all comparisons).

**Figure 5:**
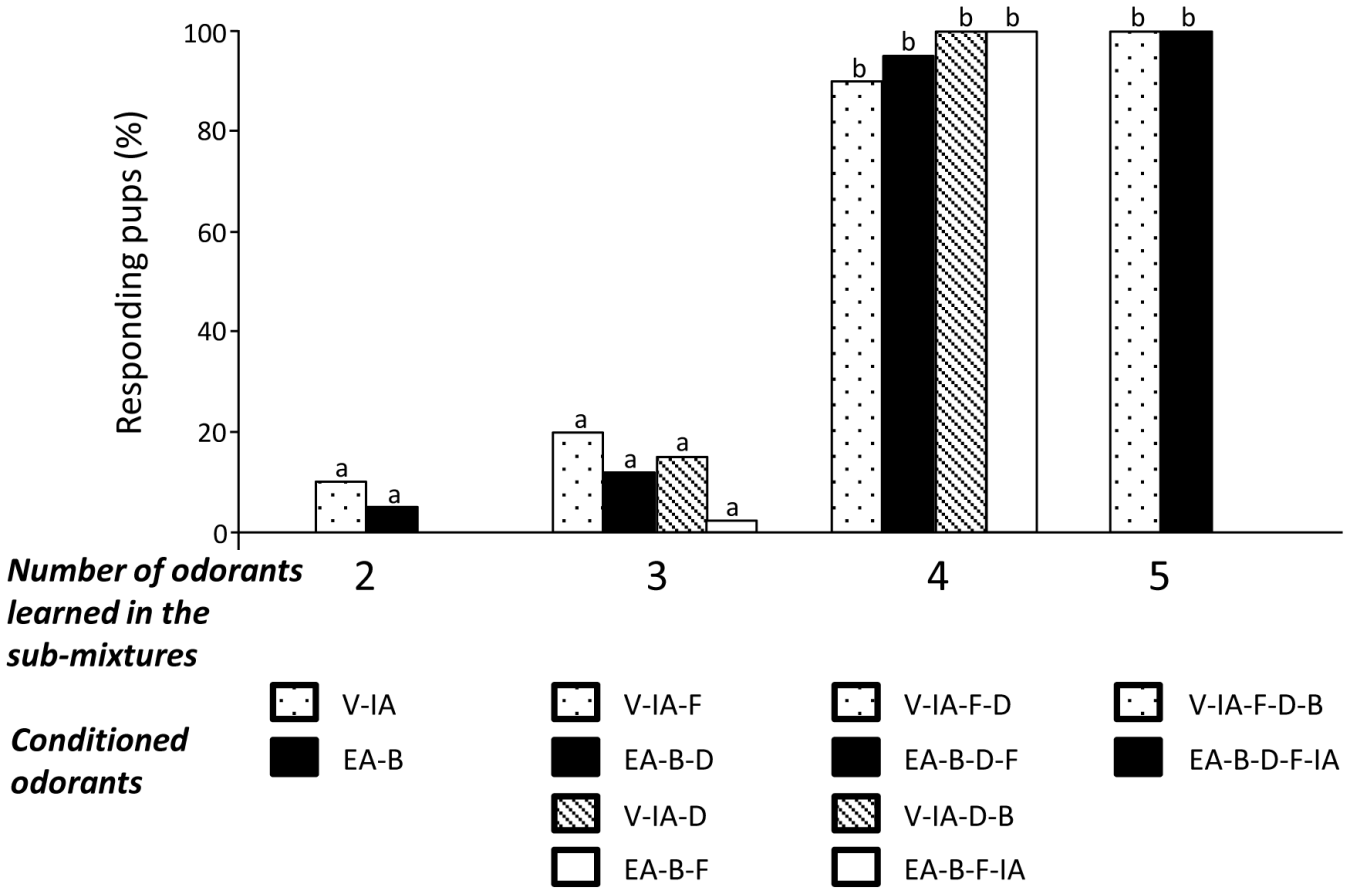
Proportions of 2-day-old rabbit pups responding in an oral activation test to the RC mixture after conditioning to sub-mixtures of 2, 3, 4 and 5 odorants that compose RC. Dotted bars represent the responsiveness to RC after conditioning to sub-mixtures containing the more RC-typical components, black bars with the less RC-typical components. Hatched and white bars illustrate the pup responsiveness to RC after conditioning to other sub-mixtures of RC components. Statistical differences are indicated by the letters on top of the bars (p<0.05).

Taken together, the results of Experiment 1 showed that newborn rabbits could generalize their behavioral response to the RC mixture after learning sub-mixtures of at least 4 odorants (Figure 5). This generalization occurred whatever the nature and odor quality of the RC elements included in the sub-mixtures used here.

### Experiment 2 - Number and nature of familiar elements required to respond to the M^V^ elemental mixture

**Figure 6:**
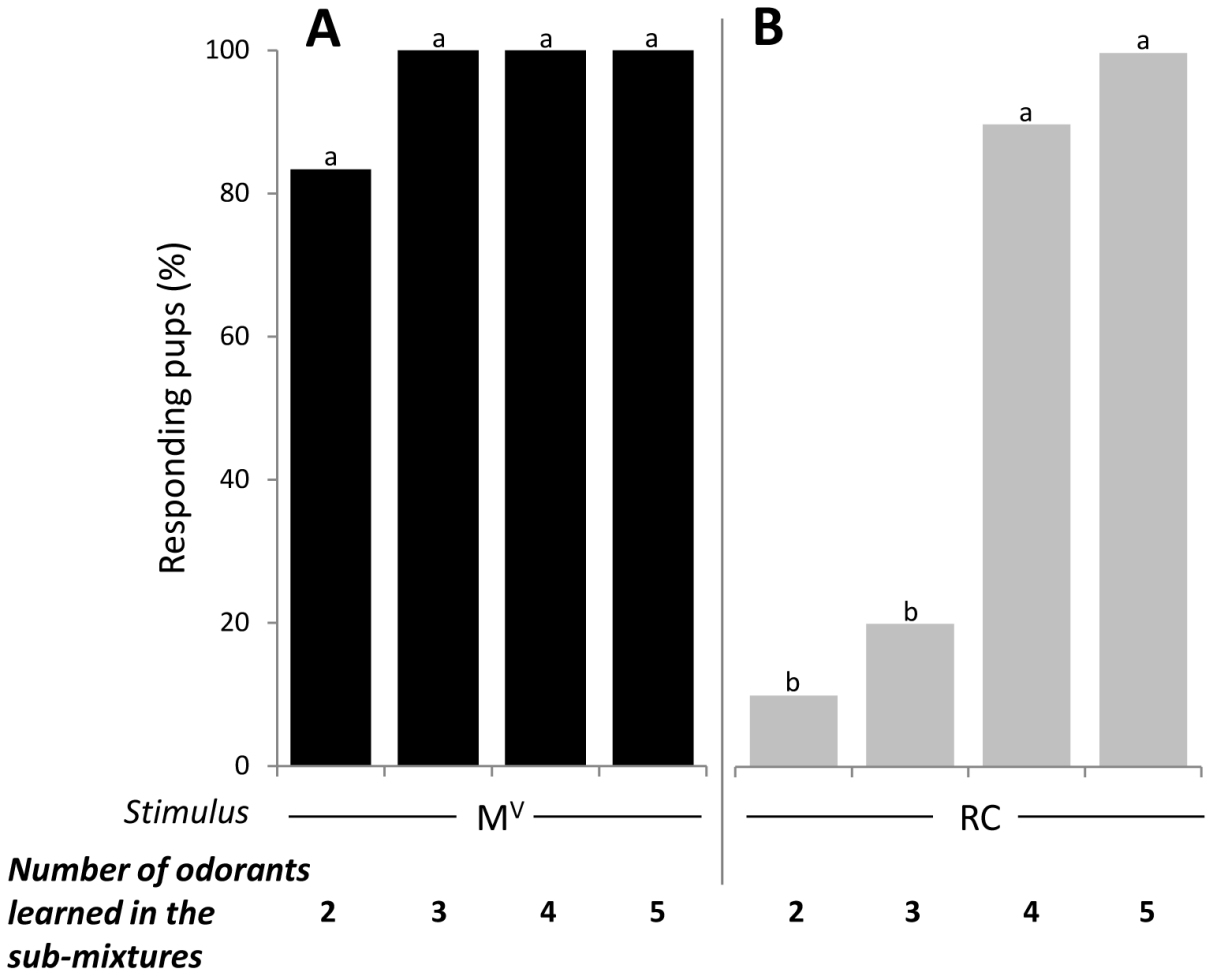
Proportions of 2-day-old rabbit pups responding in an oral activation test to (A) the M^V^ non-blending mixture (black bars, n = 12 pups per sub-mixture), or (B) to the RC blending mixture (grey bars, n = 20 per sub-mixture) after conditioning to their respective sub-mixtures of 2 to 5 components. Results for RC are the same as in Experiment 1 for the more RC-typical components (see **Figure 3**). In each graph, statistical differences are indicated by the letters on top of the bars (p<0.05).

The same procedure as in Exp. 1 was repeated with the M^V^ mixture, known to be elementally processed in newborn rabbits. Pups were conditioned to one of the following sub-mixtures of 2 to 5 components: N-L, E-P-C3H, E-P-C3H-N, E-P-C3H-N-L (4 groups of 12 pups) before testing with the sub-mixture, some of its components and M^V^. In each group, the proportion of pups responding to the conditioned sub-mixture was high (>85%). It did not differ from the responsiveness to the sub-mixture components (>90%) and to M^V^ (>80%) whatever the complexity of the learned sub-mixture (comparisons of responsiveness to M^V^ after conditioning to 2- to 5-component sub-mixtures: χ^2^ = 6.3, d.f. = 3, p>0.05) (Figure 6).

In sum, after conditioning to the sub-mixtures of the M^V^ elemental mixture used here, pups later responded to this mixture whatever the number (2 to 5) and nature of the learned components.

### Experiment 3 – Configural perception of RC sub-mixtures

**Figure 7:**
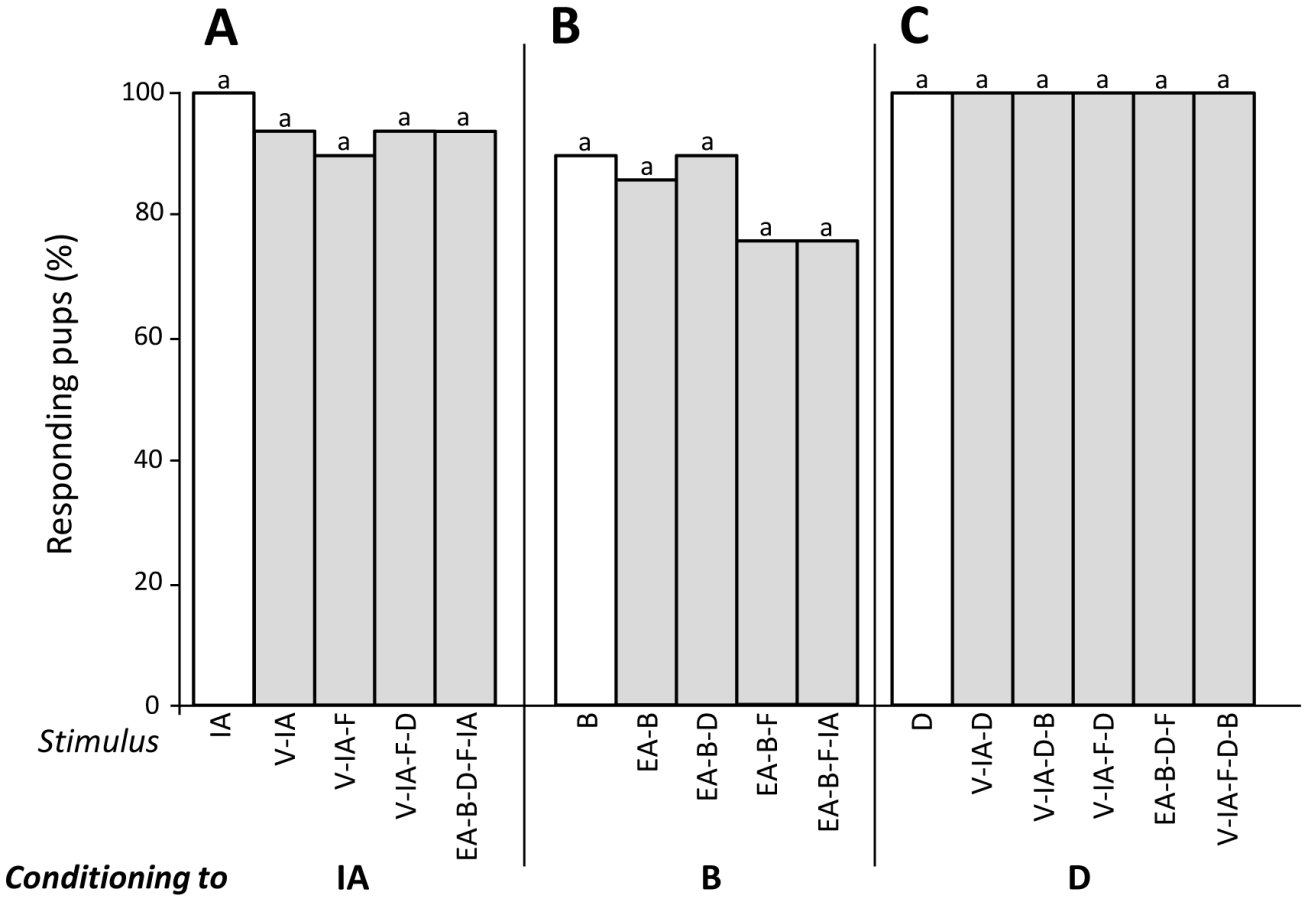
Proportions of 2-day-old rabbit pups responding in an oral activation test to the conditioned component (white bars) and the sub-mixtures (grey bars) after conditioning to one component of RC: (A) odorant IA (n = 19 pups), (B) odorant B (n = 20), and (C) odorant D (n = 35). In each group, responsiveness to the different stimuli did not differ significantly (p>0.05).

In Exp. 1, rabbit pups responded to the RC mixture after learning quaternary and quinary sub-mixtures of RC. It can be that weak configural perception of these sub-mixtures takes place and thus contributes to the generalisation of the response to the whole RC mixture. To evaluate this assumption, different groups of pups were conditioned to one single odorant before being tested to sub-mixtures. Thus, 19 pups were conditioned to odorant IA then tested to V-IA, V-IA-F, V-IA-F-D and EA-B-D-F-IA. The pups' responsiveness was high and similar (Q = 2, d.f. = 4, p>0.05) both to the conditioned stimulus (IA; 100%) and the sub-mixtures (>89%) (Figure 7A). Twenty other pups were conditioned to B then tested to B, EA-B, EA-B-D, EA-B-F and EA-B-F-IA. Again, the responsiveness was high and similar to the odorant and the sub-mixtures (>75%, Q = 5, df = 4, p>0.05, Figure 7B). Finally, 19 and 16 pups were conditioned to odorant D before being tested respectively to D, V-IA-D, V-IA-F-D, EA-B-D-F and V-IA-F-D-B, or to D and V-IA-D-B. All the pups responded to D and the sub-mixtures (Q<0.5, df = 4, p>0.05 in the two groups, Figure 7C).

Thus, after conditioning to one odorant of the RC sub-mixtures, newborn rabbits strongly responded to the sub-mixtures regardless of their content in terms of number of components. This suggested purely elemental perception of RC sub-mixtures by the pups.

## Discussion

The perception of odors results from combination of odorants that are experienced in different chemical contexts, namely among other odorants. However, it remains unclear whether experience with one or several odorants included in a sub-mixture can modulate the perception of a more complex mixture containing this odorant/these odorants. The present study aimed to determine in a newborn mammal whether the perception of complex odor mixtures varies with the quantity or the quality of previously acquired bits of information contained in these mixtures. We especially tested two types of mixtures here, one configurally and one elementally perceived (RC and M^V^ mixtures, respectively), with the hypothesis that acquired information modulates their perception differently.

After conditioning to binary or ternary sub-mixtures of RC, the pups did not generalize their response to the whole mixture during behavioral testing (Exp.1). This lack of responsiveness was not due to the inability of pups to generalize from sub-mixtures to 6-component mixtures, since after similar conditioning they strongly responded to the M^V^ mixture of same complexity (Exp.2). In fact, regarding M^V^, after conditioning to sub-mixtures of 2 to 5 odorants, the newborns are likely to have perceived the elements in the sub-mixtures and this perception was sufficient to trigger their behavioral response, always maximal to M^V^. This result confirms that M^V^ is perceived in a pure elemental way by rabbit pups (such generalization had been previously observed after conditioning to one single odorant [20]).

The present results support the hypothesis that acquired information about the components of a mixture modulates its further perception differently depending on its configural vs elemental processing. The pups' ability to generalize their response to an elemental mixture after learning one or a few components was previously observed with a binary odor mixture (AC, [9]); it is extended here to a more complex 6-component mixture (see also [20]). However, such generalization does not hold for configural mixtures. Indeed, in the case of the configurally processed binary AB or senary RC mixtures, pups did not respond to the mixture after single conditioning to only one component [8]–[13], [20]. Nevertheless, in the case of the binary AB mixture, after successive conditioning to A then B rabbit pups use the acquired bits of information to generalize their response to AB [8]. Here, we provide similar results for the complex 6-component RC mixture by showing that learning 4 odorants and more promotes the response to RC.

The fundamental difference between elemental and configural odor mixtures is that a configural mixture is processed in such a way that the combination of elements creates a configuration specific to the mixture (unique cue; [26]–[29]). Rescorla (1972) considered that the configuration emerges after conditioning to the mixture as a whole; the value acquired by a mixture after associative learning is then a combination of the associative strengths of the elements and of the strength of the configuration [30]. Conversely, Pearce [31] considered that learning is not a prerequisite for the perception of a configuration. In the present study, we assume that the RC configuration was perceived without previous learning since pups had never been exposed to the configuration before testing with RC (see also [20]); they were indeed only conditioned to sub-mixtures (Exp. 1). In this mixture, the configuration is perceived in addition to the odor qualities of the components and would prevent the response of pups when insufficient bits of information about the mixture are learned. At least three hypotheses can be proposed to explain why the responsiveness of newborn rabbits to the RC mixture changed according to the number of odorants previously learned. They concern the perception of configurations in sub-mixtures, the familiar versus unfamiliar bits of information perceived in the mixture, and the abolition of any perceptual configuration due to the acquisition of a sufficient number of elements.

*First,* the pups' responsiveness to the RC mixture after learning 4 or more of its elements could result from the perception of configurations in the sub-mixtures. However, this hypothesis has to be discarded because rabbit pups responded to all the odorants after conditioning to sub-mixtures of 4 or 5 components, demonstrating that neither overshadowing nor robust configural processing controlled their perception (Exp. 1). Moreover, pups responded to the sub-mixtures after conditioning to one of their odorants, showing that the sub-mixtures' perception was purely elemental whatever their complexity; in other words, conditioning to a RC sub-mixture was equivalent to conditioning to all the sub-mixture's components (Exp. 3).

*Second,* the pups' responsiveness to the RC mixture might depend on the ratio of familiar versus unfamiliar (learned vs unlearned) odors contained in the mixture. Indeed, rabbit pups responded to the mixture when they have learned more than 50% (four out of six) of its components. Such assumption has been proposed in the case of the weak configural AB mixture perception: the pups responded to the mixture after learning A then B since they were familiar with more than 50% of the individual information contained in the mixture (odor of A, of B and of the AB configuration), while they did not respond to AB after learning one odorant only since they knew less than 50% of the information [8]. Here, the results support the hypothesis of the permanent perception of a 7^th^ odor in the RC mixture, i.e. the RC configuration in addition to the six component odors. Then, when newborns have learned 3 or less of the components, they would not respond to RC since only 50% or less of the elemental plus configural bits of information are familiar. Conversely, they would respond after learning 4 or 5 of the odorants since they perceive a majority of familiar information in the mixture; the individual information represented by the learned elements would become then sufficient to counteract the novelty of the unconditioned odorants and of the configuration. Nevertheless, our results show that this “rule” based on the ratio of familiar/unfamiliar bits of information would not hold in the case of elementally perceived mixtures. Indeed, pups responded to the whole M^V^ mixture after learning only one among six odorants (Exp. 2). Therefore, to maintain the hypothesis for the 6-component RC mixture, another assumption is required: the “perceptual weight” of the configuration has to be higher than the perceptual weight of the elements [32]. According to Rescorla's point of view, the hypothetic weight of the configuration is equivalent to the weight of 50%, at least, of the elements, namely three elements here [26]. Considering Pearce's point of view, in the case of mixture configurally perceived the learned elements encourage responding while the configuration counteracts the response [33]. Taken together, the results obtained here could then be translated in the prospective equation for the RC and M^V^ mixtures:
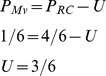
(where P  =  proportion of learned elements inducing the response to the mixture, U  =  perceptual weight of the RC configuration).

In sum, the pups would respond to M^V^ after learning one odorant (1 familiar bit of information among a total of 6, each carrying an equivalent perceptual weight) but should learn 4/6 odorants to respond to RC since the RC configuration (unique cue U) has a perceptual weight of 3/6. This equation fits also for the results obtained with the AB mixture (perceptual weight of the unique cue: 1/2) when compared to the elementally perceived AC mixture [8], [9]. It would be interesting to assess the relevance of this equation for other mixtures, at least in the newborn rabbit.

*Third*, learning at least 4 out of the 6 elements of the RC mixture could prevent the RC configural perception. Thus, when more than 50% of the elements have been learned, pups might process the RC mixture in a strict elemental manner. This hypothesis has been proposed in humans for the AB mixture after mere exposure to the A and B elements [6]. It assumes that the number of elemental vs configural information perceived in a mixture is not fixed but can change according to experience with the elements. Therefore, in contrast to our second hypothesis, it is not the familiar/unfamiliar ratio of a fixed number of information that would drive the mixture processing but the knowledge of the individual elements. Such knowledge could alter the perception of the configuration and reduce the total number of information perceived in the mixture. Here, the conditioning to 4 or 5 RC-components might prevent the perception of the RC configuration and make the perception of the mixture purely elemental. To date, animals' experience with odorants alone or in mixtures has been shown to modulate the elemental or configural perception of mixtures; however, it is usually considered that this modulation requires repetitive exposures or conditioning [13], [34]–[37]. In the present study, the effect is not the consequence of successive but of a single conditioning to a sufficient number of elements: after learning 2 or 3 odorants, the RC configuration would be still perceived by the pups; no more after learning 4 or 5 components.

Presently, it is impossible to decide between the two last hypotheses. Anyway, it clearly appears that the number of learned elements influenced the perception of the RC mixture more compared to the elements' quality. In this regard, even if we did not test all the possible RC sub-mixtures, we expect the results to be similar with the non-tested ones since those used here were carefully chosen according to their contrasted odor quality. This leads us to argue that the quality of elemental bits of information required to respond to RC do not influence the response to the mixture. In other words, in our conditions of component concentrations and proportions, no key-components seem to influence the responsiveness of newborn rabbits to the RC configural mixture. Whether this difference is due to the developmental state of the animals and/or to the specificity of the RC mixture remains to be tested.

To conclude, the present study demonstrates that newborn rabbit responsiveness to a 6-component odor mixture, initially perceived in a weak configural way, changes according to previous knowledge acquired about the elements. This underlines the high plasticity of certain odor mixtures perception, a plasticity probably related to specific brain processes occurring in the olfactory bulb and/or piriform cortex [38]–[39] even early in life [10]–[11], [40]. In the rabbit, neonates are exposed to odor cues emitted by the mother (e.g., on her abdomen, milk; [41]–[45]) including the mammary pheromone. The pheromone triggers their sucking behavior and promotes the acquisition of other odorants or mixtures [46]. Therefore, the perceptual changes observed in the present experiments are likely to occur in the natural situation of nursing and development. In these altricial newborns, in vital need to suck during the daily nursing [21], a better identification of odorants in mixtures and representation and discrimination between odorants and configuration might optimize both decision making and expression of motor actions in response to simple or complex odor signals merging from the nest or from conspecifics. More generally, deciphering the mechanisms underpinning elemental and configural perception of odor mixtures is of interest in the whole animal kingdom, including in humans.
